# The significance of PET/CT combined with machine learning models for the classification of lymphoma involvement and metastases in enlarged lymph nodes

**DOI:** 10.3389/fonc.2025.1643924

**Published:** 2025-09-30

**Authors:** Jingyi Ren, Jinbo Lu, Xun Shi, Yuexin Cheng

**Affiliations:** ^1^ Department of Nuclear Medicine, Yancheng No. 1 Peoples’ Hospital, Yancheng, China; ^2^ Department of Hematology, Yancheng No. 1 Peoples’ Hospital, Yancheng, China

**Keywords:** lymphadenopathy, lymphoma, PET/CT, SUVmax, machine learning

## Abstract

**Objective:**

Accurate differentiation between lymphoma involvement and lymph node metastasis poses significant diagnostic challenges due to overlapping imaging characteristics. This study evaluates the discriminative capacity of PET/CT metabolic profiling integrated with machine learning for nodal pathology classification.

**Methods:**

We analyzed 247 lymph nodes from patients with diffuse large B-cell lymphoma (DLBCL, n=39) and solid tumor metastases (n=46). Multivariable logistic regression identified key PET/CT biomarkers, including metabolic parameters and anatomical features. Three machine learning models—Random Forest (RF), Support Vector Machine (SVM), and Artificial Neural Network (ANN)—were trained using these predictors.

**Results:**

Lymphomatous nodes exhibited significantly elevated metabolic activity (SUV_max_ median: 16.0 vs. 10.0, P<0.001), larger short-axis diameters (13 mm vs. 11 mm, P<0.001), and concurrent splenic hypermetabolism (spleen SUV_max_ 3.1 vs. 2.8, P<0.001). The RF model demonstrated exceptional performance with an AUC of 0.942, accuracy of 93.88%, and 100% specificity, outperforming SVM (AUC = 0.850) and ANN (AUC = 0.824). Splenic metabolic parameters significantly enhanced model discrimination.

**Conclusion:**

Integration of PET/CT-derived SUV_max_ and splenic metabolic features with machine learning, particularly RF algorithms, provides a potential framework for distinguishing lymphoma-involved from metastatic nodes. This approach holds promise for optimizing biopsy decisions and refining pretreatment risk stratification in clinical oncology.

## 1 Introduction

Lymphadenopathy serves as a critical clinical manifestation across diverse pathologies, including hematologic and solid malignancies. Diffuse large B-cell lymphoma (DLBCL) constitutes 30–40% of non-Hodgkin lymphomas, with its rapid nodal enlargement directly influencing disease staging and treatment selection (1). Lymph node involvement in solid tumors typically signifies lymphatic dissemination and is associated with advanced stage and poorer prognosis. Given the distinct biological behaviors and treatment paradigms between lymphoid malignancies and metastatic epithelial cancers, accurate discrimination is clinically imperative. Beyond the initial diagnostic challenge, the clinical management of confirmed DLBCL relies heavily on prognostic stratification tools such as the International Prognostic Index (IPI) (2), which integrates clinical and laboratory parameters, and the cell-of-origin (COO) classification (3, 4), which reflects distinct molecular subtypes with therapeutic implications. It is important to emphasize that the effective application of these prognostic models is entirely contingent upon an accurate initial pathologic diagnosis, which underscores the critical clinical necessity for reliable non-invasive diagnostic tools.

Positron emission tomography/computed tomography (PET/CT) with ^18^F-fluorodeoxyglucose (FDG) has emerged as a valuable tool in oncologic imaging by providing metabolic information complementary to structural findings (5). The Standardized Uptake Value (SUV), particularly SUVmax, reflects glycolytic activity and has enhanced diagnostic accuracy in lymphoma characterization (6, 7). For instance, lymphomatous nodes often demonstrate significantly elevated SUVmax values (>15.0), outperforming size-based criteria (8, 9). Nevertheless, technical constraints persist: the partial volume effect markedly reduces diagnostic accuracy for subcentimeter lesions (<8mm), underestimating FDG uptake by 30~60% (10, 11). Additionally, 15-20% of aggressive lymphomas demonstrate unexpectedly low metabolic activity, mimicking benign conditions (12). These unresolved challenges necessitate novel approaches combining metabolic imaging biomarkers with computational analytics.

Radiomic analysis, which extracts high-dimensional, quantitative features from medical images, combined with machine learning (ML) has emerged as a promising solution to improve diagnostic accuracy (13). Several previous studies have explored its utility in differentiating lymphomatous nodes. For instance, Yang et al. (5) developed a CAD model by integrating radiomic features from both PET and CT to specifically discriminate between lymphoma involvement and lymph node metastasis in the cervix. However, the clinical translation of these approaches is often hampered by several recurring limitations (1): a reliance on single-center, retrospective cohorts, raising concerns about model generalizability (2); the development and validation using a single machine learning model (e.g., logistic regression or a solitary classifier) without comprehensive benchmarking against other established algorithms; and (3) a failure to effectively integrate metabolic parameters (e.g., from PET) with semantic morphological features (e.g., from CT) in a manner that addresses feature redundancy and collinearity through robust selection methods.

To address these gaps, this study aims to develop and validate a rigorously validated, multi-model comparative framework that leverages the strengths of both metabolic and structural imaging data. We implement and compare three distinct algorithms—Random Forest (RF), Support Vector Machine (SVM), and Artificial Neural Network (ANN)—selected for their complementary strengths. RF was chosen for its proficiency with tabular data and feature importance ranking, SVM for its effectiveness in modeling non-linear relationships with limited samples, and ANN for its capacity to automatically learn complex feature interactions. By leveraging PET/CT-derived radiomic features, our goal is to establish a non-invasive diagnostic tool to discriminate between lymphoma-involved and metastatic lymph nodes. We seek to create a tool that may assist clinical decision-making, optimize biopsy guidance, and ultimately contribute to personalized management strategies in nodal malignancies.

## 2 Materials and methods

### 2.1 Data collection and processing

Clinical and imaging data were collected from a cohort of 85 patients diagnosed with DLBCL and solid tumors at the First People’s Hospital of Yancheng City from January 2023 to October 2024, all of whom presented with enlarged lymph nodes. Among the patients, 39 were diagnosed with DLBCL, corresponding to a total of 116 lymph nodes, and 46 were diagnosed with solid tumors, accounting for 131 lymph nodes. One to four lymph nodes were selected from each patient, culminating in the examination of 247 lymph nodes overall. Additional data collected included gender, age, lymph node short axis, CT and SUV_max_ values of lymph nodes, spleen vertical diameter, spleen SUV_max_, and evidence of bone destruction. This study received approval from the Ethics Committee of the Institutional Review Board at Yancheng No.1 People’s Hospital, with informed consent being waived.

### 2.2 Inclusion and exclusion criteria

Inclusion criteria: 1) The interval between imaging examination and pathological confirmation was ≤ 2 weeks; 2) lymph node short axis > 5mm; 3) pathologically confirmed lymphoma, with lymph nodes selected adjacent to the biopsy site or larger typical masses; 4) patients with cancerous lymph nodes had a documented history of solid tumors, with pathology confirming surrounding metastases following regional lymph node dissection. Exclusion criteria: 1) Incomplete imaging data or suboptimal image quality; 2) Prior history of systemic treatment for malignant tumors before PET/CT examination.

### 2.3 Image analysis and feature extraction

All PET/CT images were first converted to a unified format (DICOM format conversion) using a dedicated Siemens workstation. Subsequently, a voxel-based standardization method was adopted to adjust the HU value range of CT images to [-1000, 400] (to eliminate interference from air and metal artifacts). PET images were subjected to attenuation correction and normalization according to the specifications of the EARL program to ensure the comparability of PET signals among different patients.

Two nuclear medicine radiologists, each with over five years of experience, independently performed measurements in a blinded manner. For each lymph node, the following were recorded:① The short-axis diameter was measured (the largest cross-section of the lymph node was selected, and the shortest diameter perpendicular to the long axis was measured); ② A region of interest (ROI) was drawn on the PET image to ensure that the ROI completely covered the lymph node area, and the SUVmax value was calculated; ③ The CT value of the lymph node was measured on the CT image (the average CT value within the ROI was taken). For the spleen, its vertical diameter was measured (the maximum vertical distance from the upper pole to the lower pole of the spleen), and the overall ROI of the Spleen measurements included vertical diameter and whole-organ SUVmax. Inter-observer discrepancies exceeding 10% were resolved through consensus. Lymph nodes were evaluated following IASLC criteria (14), with confluent clusters documented as single lesions.

### 2.4 Machine learning implementation

Three machine learning models were implemented using R version 4.1.2: Random Forest (RF): built with the randomForest package. Hyperparameters were tuned via 5-fold cross-validation, yielding ntree = 500, min.node.size = 5, and mtry = 3. Support Vector Machine (SVM): implemented using the e1071 package with a radial basis function (RBF) kernel. Grid search identified optimal parameters as cost = 1 and gamma = 0.1. Artificial Neural Network (ANN): constructed with the nnet package as a single-hidden-layer network. The final structure included 10 hidden nodes, a sigmoid activation function, a learning rate of 0.01, and was trained for 1000 iterations using gradient descent.

### 2.5 Statistical analysis

Continuous variables with non-normal distributions were analyzed using the Mann-Whitney U test, while categorical variables were assessed through chi-square tests.

To address potential overfitting and enhance the reliability of our findings given the limited sample size, we implemented two robust statistical validation techniques (1): Bootstrapping Validation: We performed 1000 bootstrap resampling iterations with replacement on the training set. In each iteration, the model was retrained on the resampled data and evaluated on the original test set to calculate performance metrics, including AUC and accuracy. The final reported performance metrics represent the mean values from these 1000 bootstrap validations, with 95% confidence intervals (CI) calculated to assess the stability and generalizability of the model (2). Permutation Test: To rigorously verify that the model’s performance was not attributable to random chance, we conducted a permutation test by randomly shuffling all sample labels (DLBCL vs. solid tumor metastasis) 1000 times. For each permutation, the model was retrained and evaluated on the test set, generating a null distribution of AUC values.

Multivariable logistic regression was employed to identify factors associated with lymphoma versus solid tumor classification. Model performance was evaluated using confusion matrix-derived metrics including accuracy (Acc), sensitivity (Sens), specificity (Spec), positive predictive value (PPV), negative predictive value (NPV), and F1 scores. Discriminatory capacity was quantified by the area under the receiver operating characteristic curve (AUC), with inter-model AUC comparisons performed using DeLong’s test. Statistical significance was set at p < 0.05, with significance levels denoted as * *p* < 0.05, ** *p* < 0.01, and *** *p* < 0.001.

## 3 Results

### 3.1 Patient characteristics

The demographic and clinical characteristics of the study population are detailed in **Table 1**. Our cohort included 39 patients with DLBCL and 46 with solid tumor metastases. The groups were well-matched in demographic composition, with no significant differences in gender distribution (DLBCL: 20 males, 19 females; solid tumors: 27 males, 19 females; *p* = 0.423) or median age (DLBCL: 67 years; solid tumors: 65 years; *p* = 0.316).

**Table 1:** Clinical characteristics of patients in the DLBCL and solid tumors groups.

Characteristic	DLBCL (n=39)	Solid tumors (n=46)	*p* value
Age,median(IQR),years	66 (58~71)	66 (61~72)	0.120
Gender			0.850
Female	17 (44.0%)	21 (46.0%)	
Male	22 (56.0%)	25 (54.0%)	
Lymph node short axis diameter (IQR),mm	13 (10~17)	11 (9~14)	<0.001
Lymph node SUV_max_	16 (10~25)	10 (7~13)	<0.001
Lymph node CT,HU	35 (30~40)	37 (32~42)	0.180
Spleen vertical diameter (IQR),mm	80 (63~110)	65 (61~79)	<0.001
Spleen SUV_max_	3.1 (2.5~5.1)	2.8 (2.2~3.3)	<0.001
Bone destruction			0.340
Yes	13 (33.0%)	11 (24.0%)	
No	26 (66.0%)	35 (76.0%)	
Pathology			<0.001
breast	0 (0.0%)	2 (4.3%)	
cervical	0 (0.0%)	1 (2.2%)	
colon	0 (0.0%)	3 (6.5%)	
CUP	0 (0.0%)	4 (8.7%)	
DLBCL	39 (100.0%)	0 (0.0%)	
liver	0 (0.0%)	4 (8.7%)	
lung	0 (0.0%)	23 (50.0%)	
melanoma	0 (0.0%)	1 (2.2%)	
pancreas	0 (0.0%)	1 (2.2%)	
rectum	0 (0.0%)	1 (2.2%)	
stomach	0 (0.0%)	5 (11.0%)	
thyroid	0 (0.0%)	1 (2.2%)	

HU, Hounsfield Units; IQR, interquartile range; CUP, Cancer of Unknown Primary.

**Figure 1:**
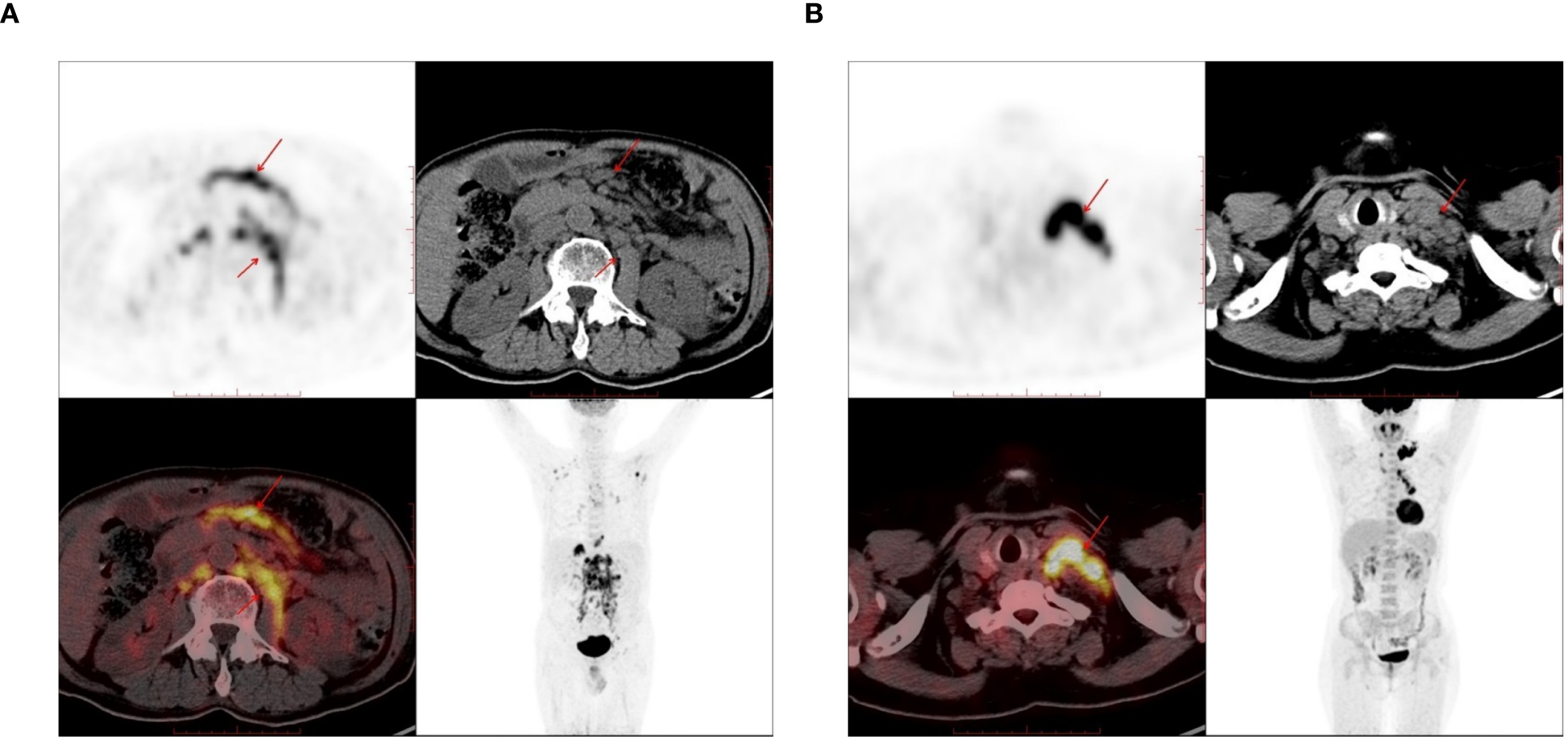
The PET-CT images used in the study. **(A)** 72-year-old male, there are enlarged lymph nodes in the abdominal cavity and retroperitoneum, which were pathologically diagnosed as diffuse large B-cell lymphoma (DLBCL). **(B)** A 61-year-old female, there are enlarged lymph nodes in the left supraclavicular region, which was pathologically diagnosed as lung adenocarcinoma.

A total of 116 lymphoma-involved nodes and 131 metastatic nodes were analyzed. Significant disparities in PET/CT biomarkers were observed between the two groups. Specifically, lymphoma-involved nodes demonstrated markedly higher metabolic activity, with a significantly elevated median SUVmax (21.4 vs. 8.9; *p* < 0.001) and larger short-axis diameter (3.2 cm vs. 1.8 cm; *p* < 0.001). Systemic involvement in the DLBCL group was further evidenced by a higher rate of splenomegaly (43.6% vs. 8.7%; *p* < 0.001) and significantly increased splenic SUVmax (4.1 vs. 2.3; *p* = 0.002). In contrast, conventional CT morphological features such as the presence of necrosis or calcification did not differ significantly between groups. Bone destruction was rare in both cohorts and not statistically different. Representative PET/CT manifestations of lymphomatous and metastatic nodal involvement are illustrated in **Figure 1**. These findings underscore the value of PET-derived metabolic parameters, particularly nodal SUVmax and splenic metrics, as robust discriminators between DLBCL and metastatic nodal disease, supporting their inclusion as key features in subsequent predictive modeling.

### 3.2 Multifactorial logistic analysis

**Figure 2:**
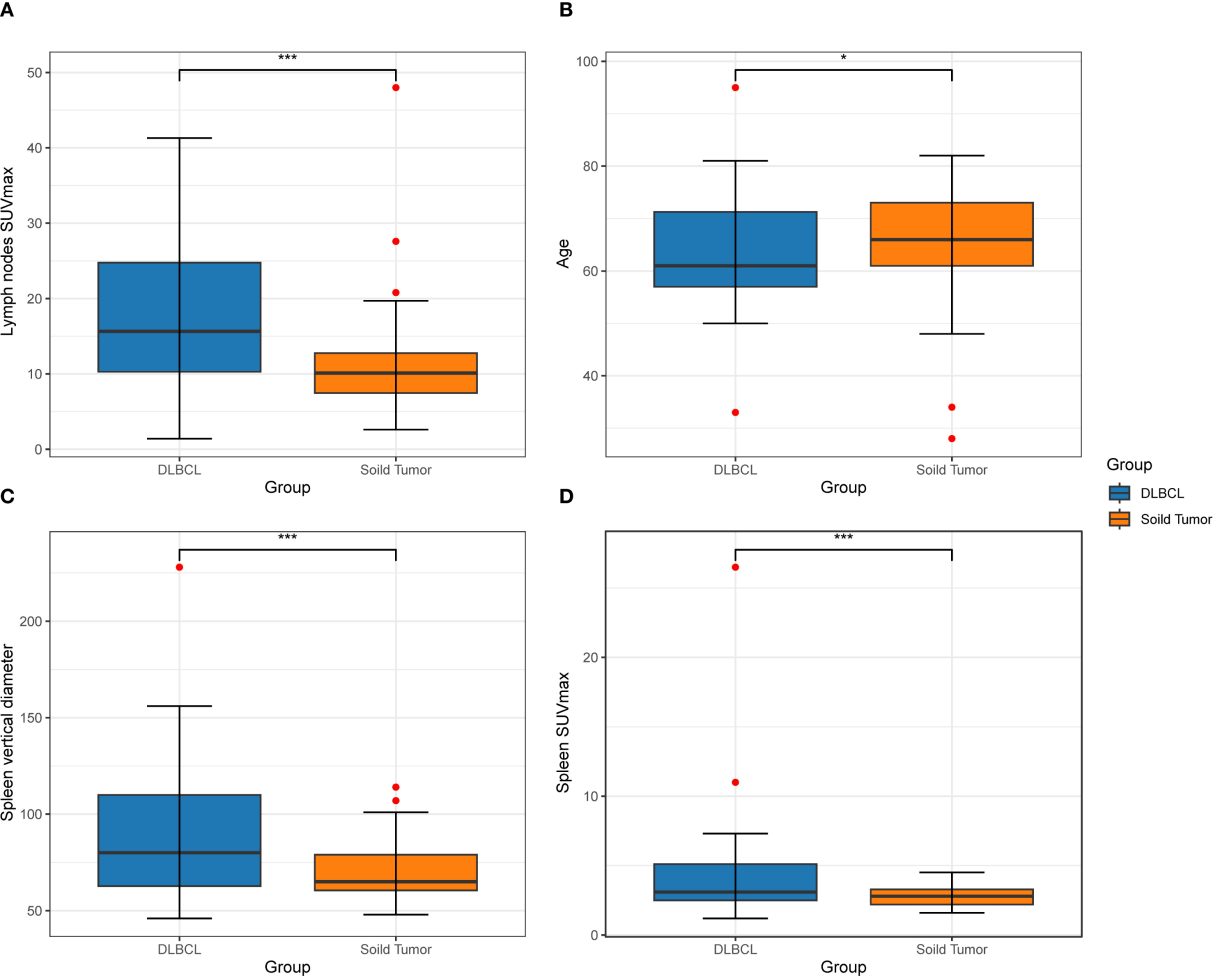
A box diagram depicts the disparity in two groups among patients in lymph nodes SUV_max_ **(A)**, age **(B)**, vertical diameter of the spleen **(C)**, and spleen SUV_max_ (_D_).

Multivariable logistic regression identified several independent predictors of lymphoma involvement in enlarged lymph nodes, as detailed in **Table 2**. The box plots of lymph nodes SUV_max_, age, spleen vertical diameter and spleen SUV_max_ are shown in **Figures 2A–D**. PET-derived metabolic parameters as dominant predictors of lymphoma involvement, with lymph node SUV_max_ demonstrating the strongest association (OR = 1.119, 95%CI 1.063~1.186, *p* < 0.05). Splenic characteristics showed systemic diagnostic relevance, particularly splenic SUV_max_ (OR = 1.830, *p* < 0.05). Notably, osseous involvement exhibited an inverse relationship with lymphoma probability (OR = 0.174, *p* < 0.05), suggesting potential differential bone remodeling patterns between lymphoma and metastatic lesions.

**Table 2:** Multivariable logistic regression analysis of lymphomatous involvement.

Clinical variables	OR	95%CI	*p* value
Age	1.039	1.006 ~ 1.075	0.023
Male	0.779	0.378 ~ 1.593	0.500
“Lymph node short-axis diameter (mm)	1.048	0.977 ~ 1.129	0.200
Lymph node SUV_max_	1.119	1.063 ~ 1.186	<0.001
Lymph node CT (HU)	0.977	0.946 ~ 1.009	0.160
Spleen vertical diameter (mm)	1.028	1.010 ~ 1.049	<0.001
Spleen SUV_max_	1.830	1.245 ~ 2.813	0.003
Bone destruction	0.174	0.000 ~ 0.535	0.003

OR, Odds ratio; CI, confidence interval.

### 3.3 Establishment and validation of machine learning models

**Figure 3:**
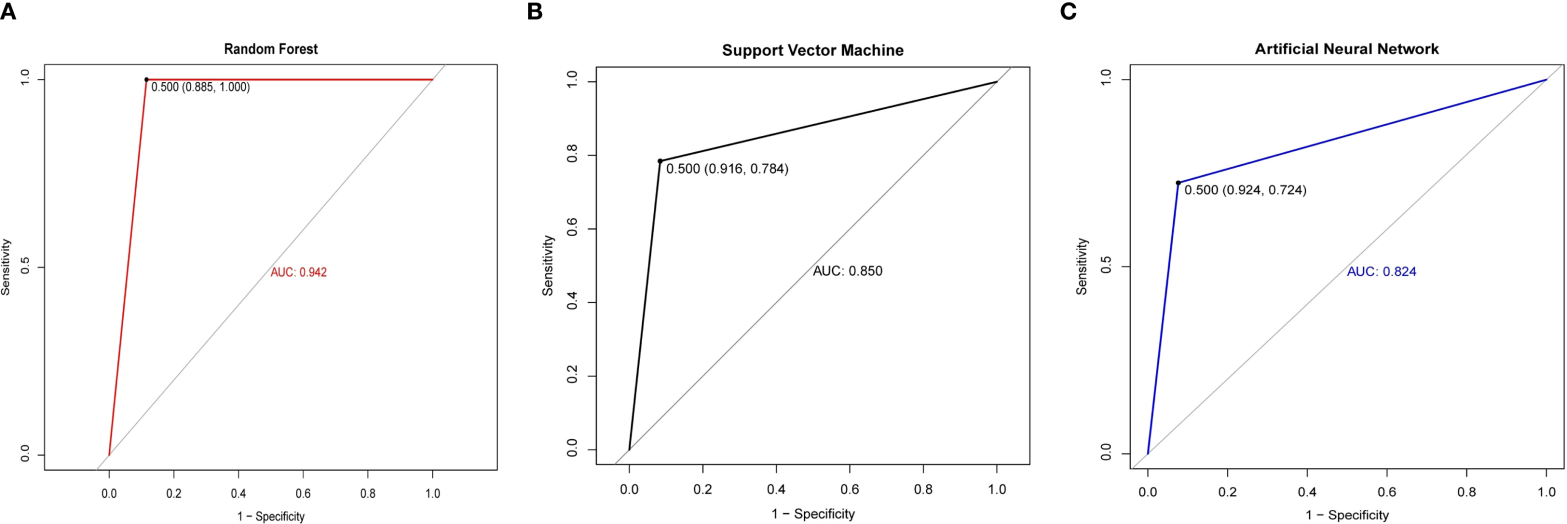
ROC curves of different models between the cancerous lymph nodes and lymphomatous lymph nodes. **(A)** ROC curves of the Random Forest. **(B)** ROC curves of the Support Vector Machine. **(C)** ROC curves of the Artificial Neural Network.

To mitigate overfitting and enhance the reliability of our findings, we employed bootstrapping and permutation tests for statistical validation. The significant predictors identified—including age, splenic vertical diameter, and metabolic biomarkers—were incorporated into three machine learning architectures: Random Forest (RF), Support Vector Machine (SVM), and Artificial Neural Network (ANN). Comparative ROC analysis revealed significant performance disparities among models (**Figure 3**), with the RF algorithm achieving superior discriminatory capacity (AUC = 0.942) compared to SVM (AUC = 0.850) and ANN (AUC = 0.824). As detailed in **Table 3**, the RF model demonstrated enhanced classification metrics including accuracy (93.88% vs 83.00% ~ 85.43%), specificity (100.00% vs 72.00% ~ 78.45%), and F1-score (0.94 vs 0.77~0.87), while maintaining balanced sensitivity (88.46%) and perfect positive predictive value (100.00%). These results affirm the ability of the RF model to effectively integrate multimodal PET/CT biomarkers for distinguishing between lymphomatous and metastatic involvement in pathologically enlarged lymph nodes.

**Table 3:** Comparative performance of machine learning architectures.

Model	Acc(%)	Sens(%)	Spec(%)	PPV(%)	NPV(%)	F1-score
Random Forest	93.88	88.46	100.00	100.00	88.46	0.94
Support Vector Machine	85.43	91.60	78.45	82.76	89.22	0.87
Artificial Neural Network	83.00	87.79	72.00	68.45	79.75	0.77

## 4 Discussion

This study demonstrates that radiomics features, particularly SUV_max_, serve as pivotal biomarkers for differentiating lymphomatous involvement from metastatic disease in enlarged lymph nodes. The biological basis of this discrimination lies in the distinct glucose metabolism patterns between these entities (15). Lymphoma cells, particularly germinal center B-cells in DLBCL, exhibit markedly accelerated glycolytic metabolism driven by intracellular hexokinase activity, resulting in pronounced 18F-FDG trapping and consequently elevated SUVmax values (16, 17). This metabolic reprogramming is frequently associated with activation of oncogenic pathways such as PI3K/Akt and MYC, which further enhance glycolytic flux and sustain the aggressive phenotype of DLBCL (18, 19). MYC, in particular, functions as a master regulator of glycolysis and other metabolic processes, exacerbating the shift toward increased glucose uptake and utilization that characterizes cancer metabolism (8). In contrast, metastatic lymph nodes from epithelial tumors typically demonstrate relatively constrained FDG avidity, reflecting their origin from cancers with less pronounced adaptations to aerobic glycolysis (20).

Consistent with these biological mechanisms, our data revealed significantly higher median SUVmax values in lymphomatous nodes compared to metastatic nodes (18.4 vs. 9.1, *p* < 0.001). This metabolic divergence was further reflected in systemic manifestations—DLBCL patients exhibited both splenomegaly (vertical diameter: 80 vs. 65 mm) and elevated splenic metabolic activity (SUVmax: 3.1 vs. 2.8), suggesting a whole-body metabolic dysregulation distinct from the localized nodal involvement characteristic of solid tumor metastases. Multivariable analysis confirmed the diagnostic superiority of SUVmax over conventional CT parameters. Machine learning models effectively captured complex nonlinear interactions between metabolic and morphological features, with the Random Forest (RF) algorithm achieving 88.46% sensitivity and 100% specificity by integrating SUVmax with lymph node short-axis diameter and CT attenuation values. This performance demonstrates the clinical viability of a multimodal biomarker approach for nodal characterization.

Among the three machine learning models developed for classifying lymphomatous versus metastatic lymph nodes, RF demonstrated optimal performance with an AUC of 0.942 and accuracy of 93.88% in our cohort. The RF algorithm showed superior discriminative capacity across all metrics—accuracy, specificity, and F1-score (0.94 vs. 0.77-0.87)—while maintaining balanced sensitivity and perfect positive predictive value. Notably, RF’s exceptional specificity in perfectly differentiating metastatic nodes (100.00%) contrasted sharply with SVM’s 78.45% and ANN’s 72.00% specificity. This precision advantage was further substantiated by RF’s maximal PPV/NPV concordance (100.00%/88.46%), significantly outperforming SVM (82.76%/89.22%) and ANN (68.45%/79.75%). RF’s robustness can be attributed to its ensemble learning framework, which mitigates overfitting and enhances generalization through aggregating predictions from multiple decision trees. This characteristic proves particularly valuable in clinical settings where variability in patient presentations and imaging artifacts may complicate diagnostic accuracy. The strength of RF in handling complex biomedical data is further supported by a recent study that employed this algorithm to differentiate between IgG4-related ophthalmic disease and orbital mucosa-associated lymphoid tissue lymphoma, achieving an AUC of 0.983 (21). This independent validation reinforces the value of ensemble methods like RF in discriminating complex oncological conditions based on multidimensional data.

Our study integrated clinical data with PET/CT imaging parameters to improve the etiological determination of enlarged lymph nodes without incurring additional medical costs. This radiomic-guided triage strategy aligns with emerging evidence supporting precision oncology paradigms, particularly in avoiding unnecessary invasive procedures (22, 23). However, it is important to acknowledge the limitation posed by our sample size, a common challenge in radiomics research investigating specific clinical questions. To mitigate potential overfitting and enhance the robustness of our findings, we employed rigorous validation strategies including bootstrapping and permutation tests. The bootstrap validation results (average AUC: 0.938, 95% CI: 0.892-0.971) demonstrated remarkable consistency with our initial test performance (AUC: 0.942), indicating stable model performance. Furthermore, the permutation test confirmed that our model’s predictive capability was statistically significant (p < 0.001) and not attributable to chance correlations (24).

The integration of clinical data with imaging parameters enriches the feature set for machine learning models and enables a more comprehensive assessment of lymph node pathology. This multimodal approach aligns with the growing trend in precision medicine, where individualized patient management strategies are informed by diverse data sources. RF’s achievement of perfect positive predictive value indicates its potential utility in clinical decision-making, particularly in distinguishing between lymphoma and metastatic involvement, which carries significant implications for treatment planning and prognostication. Future studies with larger, multi-center cohorts are warranted to further validate these findings and explore the potential complementary value of established clinical indices such as the International Prognostic Index, which could not be comprehensively evaluated in the present study due to limitations in data completeness.

## 5 Limitation

While this study offers novel insights into the application of machine learning for lymphoma classification using PET/CT, several limitations should be acknowledged. First, the single-center, retrospective design and limited sample size may constrain the generalizability of our findings, particularly with respect to rare lymphoma subtypes and oligometastatic disease. Second, the current binary classification framework does not account for other causes of lymphadenopathy, such as granulomatous or inflammatory conditions, highlighting the need for validation across more diverse etiologies and multi-institutional cohorts. Third, technical limitations related to partial volume effects—especially in subcentimeter lymph nodes (<1 cm)—may lead to underestimation of glycolytic activity and affect feature quantification (25). In conclusion, although this work provides preliminary evidence supporting the use of machine learning for tumor diagnosis based on PET/CT radiomics, further large-scale, prospective, and multicenter validation is essential to confirm its clinical utility and facilitate widespread adoption.

## 6 Conclusion

Distinguishing between lymphomatous lymph nodes and cancerous lymph nodes remains a significant diagnostic challenge in clinical practice. This study demonstrates that integrating radiomic features from PET/CT with advanced machine learning algorithms can significantly enhance the accuracy and efficiency of this differentiation. Our findings support the potential of such computer-aided systems to inform clinical decision-making, guide biopsy planning, and contribute to personalized treatment strategies. Future research involving larger, multi-center cohorts will be essential to further validate and refine these models, ultimately paving the way for their translation into routine clinical care and improved patient outcomes.

## Data Availability

The raw data supporting the conclusions of this article will be made available by the authors, without undue reservation.
